# Managing Shifting Visitor Restrictions in Hospitals during the COVID-19 Pandemic from National Authority Level to Charge Nurses' Practice: A Descriptive Study

**DOI:** 10.1155/2024/1393767

**Published:** 2024-04-27

**Authors:** Anne Sophie Ågård, Hanne Mainz, Tina Wang Vedelø, Gitte Susanne Rasmussen, Merete Gregersen

**Affiliations:** ^1^Department of Intensive Care, Aarhus University Hospital, Aarhus, Denmark; ^2^Department of Public Health, Research Unit for Nursing and Healthcare, Aarhus University, Aarhus, Denmark; ^3^Research Centre for Patient Involvement, Aarhus University and Central Denmark Region, Aarhus, Denmark; ^4^Research Centre of Emergency Medicine, Aarhus University Hospital, Aarhus, Denmark; ^5^Clinical Nursing Research Unit, Aalborg University Hospital, Aalborg, Denmark; ^6^Department of Neurosurgery, Aarhus University Hospital, Aarhus, Denmark; ^7^Department of Dermatology and Venereology, Aarhus University Hospital, Aarhus, Denmark; ^8^Department of Geriatrics, Aarhus University Hospital, Aarhus, Denmark

## Abstract

**Introduction:**

Little is known about how shifting hospital visitor restrictions issued by national health authorities were communicated, managed, and adapted by hospital charge nurses during the COVID-19 pandemic.

**Aims:**

To describe the shifting visitor restrictions and the passing on of restrictions from the national authority level to charge nurses and secondly describe charge nurses' management of the restrictions and their challenges when enforcing them.

**Methods:**

The study consisted of a document analysis and a cross-sectional survey including open-ended questions. Descriptive statistics and qualitative content analysis were used. The survey was distributed online to 88 charge nurses in somatic units in a Danish university hospital from March 2020 to April 2021.

**Results:**

Restrictions were communicated from national authority level in an effective administrative cascade. The charge nurses led their enforcement in each unit. In total, 71 charge nurses (81%) responded to the survey. For 70%, the wording of the restrictions was clear, while 31% found them challenging to handle. On a weekly or daily basis, 68% of the charge nurses deviated from the restrictions. They identified both upsides and downsides to the absence of relatives. Communication, collaboration, and leadership were experienced as key tools in the ongoing processes of adapting to shifting restrictions.

**Conclusion:**

During this severe health crisis, essential information was passed on through well-defined management levels in an effective communication pathway. Charge nurses and their professional values were challenged when balancing shifting national restrictions against individual needs of patients and relatives. *Implications for Nursing Management*. Charge nurses serve as vital intermediaries between national authorities and frontline nursing practice in managing shifting visitor restrictions during a pandemic. Their experiences can contribute to further qualifying nurse managers' considerations when designing family-centred hospital visitor policies for the future. Also, they may strengthen the handling of future sudden major organizational changes.

## 1. Background

In hospitals around the world, the Coronavirus Disease 2019 (COVID-19) pandemic caused by the Severe Acute Respiratory Syndrome Coronavirus 2 (SARS-CoV-2) has led to visitor restrictions to prevent the spread of the virus. Internationally, healthcare organizations have implemented policies and guidelines to restrict visitors' access to hospitals.

Hospital visitor restrictions have been a source of suffering and distress for all parties involved [1–6]. The absence of relatives is one of the most commonly identified factors that increase inpatients' anxiety when hospitalised with COVID-19 [3]. Furthermore, feelings of social isolation from close relatives and a lack of psychosocial support have been reported among COVID-19 inpatients [7]. For family members of both COVID-19 patients and other hospitalised patients, worry, anxiety, and uncertainty are common, and they have reported an increased need for information from care providers during the pandemic [8]. Relatives play an important role for individuals suffering from acute or chronic illness for shorter or longer periods of time, and the informal caregiving provided by relatives has been described as the backbone of care provision [9]. In addition to offering their affection and support, relatives sometimes take on more demanding tasks or responsibilities related to the nursing care, treatment, or rehabilitation of their loved one [10, 11], highlighting relatives' roles as important partners in many areas of patient care.

Prior to the pandemic, Danish hospitals generally had liberal visiting policies, acknowledging the ties between patients and their relatives and the important supportive role of relatives during hospitalisation and beyond. Consequently, in 2018, the board of directors at the 850-bed Aarhus University Hospital, where the present study was conducted, announced the end of fixed visiting hours. Therefore, when visitor restrictions were imposed upon all Danish hospitals in March 2020, this represented a historic break with a strong tradition among Danish healthcare professionals (HCPs) of welcoming and involving patients' relatives. During the pandemic, the restrictions have been loosened and tightened several times, challenging HCPs to stay updated and to deal with the implications of these restrictions for patients, relatives, and staff. At times, enforcing the shifting restrictions has been a challenging task for frontline nurses [12].

To manage the restrictions, charge nurses have played a vital role in this process. In a Danish study, hospital charge nurses with formal management education and leaders with more than five years of experience more effectively managed the COVID-19 situation [13]. However, shifting visitor restrictions during the pandemic may have complicated charge nurses' management efforts. Therefore, we aimed firstly to describe how the shifting visitor restrictions were passed on from the national authority level to the charge nurses in a university hospital and secondly to describe the charge nurses' efforts to manage the shifting restrictions in bed wards and outpatient clinics and their challenges doing so.

## 2. Design and Methods

### 2.1. Design

The study was conducted in two parts, according to its aims. Part 1 was a document analysis describing the shifting visitor restrictions in the process from decision making to their operationalisation in clinical practice. Part 2 was a cross-sectional survey exploring charge nurses' efforts to manage the shifting restrictions and their challenges doing so. We followed the CROSS guidelines for surveys [14].

### 2.2. Methods

#### 2.2.1. Part 1: Document Analysis

The document analysis was inspired by the guidelines for document analyses (CARDA), including recommendations for description of the data collection procedure and a data analysis [15].

*(1) Legislative Context*. In a Danish legislative context, the Danish Ministry of Health determines the overall regulatory and supervisory functions of national healthcare, while the five Danish regions are primarily responsible for the administration of Danish public hospitals [16, 17]. The Danish Epidemic Act, which dates to the plague epidemic in 1665, authorizes the Ministry of Health to issue restrictions to prevent infectious diseases from spreading in or outside of Denmark [18]. Under this act, managing pandemics was considered a public area of responsibility and applied to the entire population [19]. The Epidemic Act takes precedence over all other national legislation, except the Danish Constitution [20, 21].

*(2) Data Collection*. A comprehensive collection of publicly available electronic documents was retrieved by the researchers HM and TWV between March 2020 and June 2021. The data comprised various sources, including public health policy documents, institutional formal letters, and minutes of meetings of the regional council and hospital administration. The data were retrieved from national authorities, hospital websites, intranet pages, and institutional instructions and files. To ensure the thoroughness of the document collection, the process was extended to references and attachments associated with the primary materials. Six key words were used in the search for documents: Visitor restrictions, hospital visitor restrictions, relative, coronavirus, COVID-19, SARS-CoV-2, and pandemic. All documents containing instructions or orders regarding restricting relatives' access to public and private hospitals were included in the analysis.

*(3) Data Analysis*. The analysis procedures involved systematic reviewing of all collected data sources. To describe the information paths among authorities and hospital organizations in the enforcement of the visiting restrictions, a systematic content analysis of the documents was conducted in two phases [22]. The initial phase aimed to describe the information cascade in the decision-making processes related to hospital visitation restrictions. This included identifying the individuals or organizations responsible for determining, authoring, and managing the restrictions, as well as the recipients to whom these directives were forwarded and again passed on to finally be implemented in practice. The second phase of the content analysis focused on tracking changes and developments over time regarding the visitor restrictions, focusing on how they were clarified, loosened, or tightened in both the first and second waves of the pandemic.

#### 2.2.2. Part 2: Cross-Sectional Survey

*(1) Questionnaire Development and Validation*. Based on relevant literature and experience, the five authors developed a questionnaire. Besides questions about background characteristics of the respondents, the questionnaire included five multiple-choice questions:

1. How did you as leader get information about current visitor restrictions? (7 options)
2. How did you pass on the information about visitor restriction to your staff? (5 options)
3. How clear was the wording of the visitor restrictions to understand? (3-point Likert scale)
4. How did you as leader experience handling the visitor restrictions in practice? (3-point Likert scale)
5. How often have you deviated from visitor restrictions paying regard to patients and their relatives? (5-point Likert scale)

Also, the questionnaire included three open-ended questions allowing charge nurses to elaborate on selected aspects of their experiences or highlight important issues that were not otherwise addressed in the study:

- Did you have any doubts about visitor restrictions, and if so, how were you able to clarify your doubts?
- As charge nurse, is there anything you would do differently if a similar situation should occur in the future?
- Is there anything else you would like to add?

The questionnaire was validated by cognitive interviewing [23] of four charge nurses representing the target group. They were asked individually to complete the questionnaire with one of the authors by their side. By using the think aloud principles [24], the charge nurses were asked about their understanding of the questions, considerations about the answers, and the relevance of the questions. The charge nurses found that some questions could be answered differently, depending on whether they were thinking about the first or second wave of the pandemic. Therefore, three of the multiple-choice questions were divided into sections concerning the first and second waves, respectively. In addition, some questions were revised for clarification. The revised questionnaire was then converted into an electronic version, which again was pretested by two charge nurses; no further revision was needed. After adjusting the visual layout, a final version to be used in the study was accepted.

*(2) Study Population and Sampling*. In April and May 2021, the electronic questionnaire was emailed to 88 charge nurses in the hospital's somatic wards, including bed wards, outpatient clinics, short-term units, same-day surgery units, intensive care units, and mixed units. To further create attention to the survey, when launching it, the authors also announced the survey through their hospital networks. After two weeks, a reminder was emailed to those nurses who had still not responded. The online survey was created using Research Electronic Data Capture (REDCap), a safe software database for research studies, which does not allow answering the questionnaire twice [25].

*(3) Analysis of Survey Data.*

 
*Quantitative Data*. The quantitative data were transferred from REDCap to Stata version 17.0 (StataCorp LLC, College Station, TX). The charge nurses' characteristics were analyzed using simple descriptive statistics. Each of the five multiple-choice questions was calculated as percentages. No imputations were made on missing data.
 
*Qualitative Data*. The qualitative analysis of the responses to the three open-ended questions was inspired by Graneheim and Lundman's content analysis method [26]. Initially, the responses were read several times by all the authors, to get an overall understanding of the data, while making individual notes of impressions and first analytic ideas. Next, authors ASÅ, TWV, and GSR developed initial codes by systematically identifying and labelling meaning units from the text that seemed to capture key aspects. Concurrently, they identified categories in the data and summarized illustrative analytic points. The qualitative data collected through the electronically administered questionnaire did not allow us the possibility to go back and ask the respondents to elaborate on their comments. Consequently, the focus of the analysis was primarily on a manifest level (what the text says) rather than on a latent level (what the text talks about). For each of the three open-ended questions, the analytic process was repeated. During the entire process of analysis, attention was paid to the fundamental importance of researcher reflexivity, i.e., a researcher's critical self-reflection about her or his own personal background, preferences and preconceptions, and their influence on the study [27]. To further strengthen reflexivity and increase the credibility of the findings, in the final steps of the process, authors HM and MG were involved for new rounds of reflective discussions until consensus among all authors was reached on the final description of the findings [28].

### 2.3. Ethics

The study was approved by the board of directors at Aarhus University Hospital. According to the Danish Ethical Committee Law § 14, subsection 2, ethical approval by the Central Denmark Region Committees on Health Research Ethics or by the Danish Committee on Health Research Ethics was not required for this type of study. Written permission to e-mail the questionnaire to the departments' charge nurses was obtained from the senior nurse manager in each hospital department. Information about the study, voluntary participation, and anonymity was provided in the initial e-mail to the charge nurses as well as in the introductory text of the questionnaire. Completing the survey was considered consent to participation. When completing the survey in REDCap, all participant information was anonymised, preventing any direct or indirect identification of individual participants. The study was conducted according to the principles of the Declaration of Helsinki [29].

## 3. Results

### 3.1. Part 1: Document Analysis

**Figure 1:**
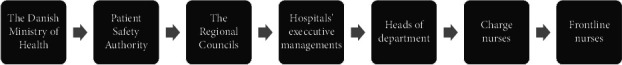
The administrative cascade for information on visitor restriction.

The Epidemic Act was the legal framework for Denmark's lockdown, which was proclaimed on 18 March 2020 by the Danish Prime Minister. The comprehensive societal restrictions were based on a precautionary approach to prevent the spread of the virus [30]. The document analysis showed that, when changes were decided by the Ministry of Health, restrictions were carried into effect via an administrative cascade (Figure 1).

The Danish Patient Safety Authority instructed the Regional Councils in the five Danish regions to issue orders to restrict relatives' access to public and private hospitals. From the Regional Councils, the restrictions were passed on to hospitals' boards of directors and from there to all hospital heads of department. They, in turn, communicated the restrictions to their charge nurses. At the endpoint of the administrative cascade, frontline healthcare professionals carried the restrictions into effect in their clinical practice. In addition, hospital administrations took steps to continuously update the regional and hospital-based electronic guidelines on visitor restrictions and to post information about restrictions on the hospital's web page.

**Figure 2:**
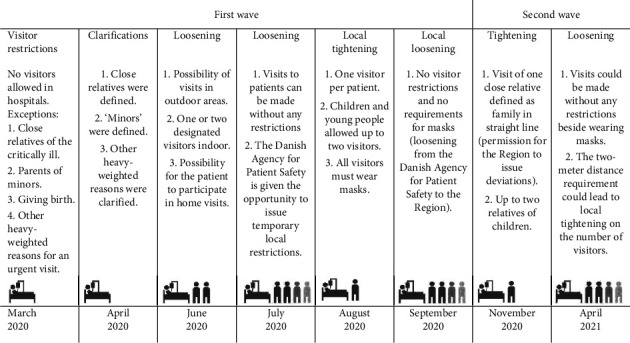
Changes in visitor restrictions from March 2020 to April 2021.

The visitor restrictions were clarified, loosened, or tightened eight times between March 2020 and April 2021 (Figure 2), reflecting the fluctuating infection rates. In March 2020, in the early days of the first wave, Denmark went into lockdown, which led to a strict no-visiting policy at the hospitals. One month later, this general protective measure was clarified by defining the term “close relative” as family in a straight line or, according to a specific assessment, a close relative. Furthermore, the term “minors” was defined as a child under the age of 18. Also, if a patient suffered from cognitive impairment, it was considered a critical reason for still allowing family visits.

Due to a decrease in infection rates in June 2020, two relatives were allowed to visit all patients, and in July, all restrictions were lifted. In cases of future local increases in infection rates, temporary restrictions applying to the affected regions only could be issued. Due to a local increase in the infection rate in August 2020, only one visitor per patient was allowed at our hospital. One month later, the local infection rate was acceptable, and consequently, the visitor restrictions were lifted. Still, all visitors were instructed to wear a face mask during their entire hospital visit. In November 2020, the second wave hit Denmark, and once again the visitor restrictions were tightened, allowing visits from one close relative only. The high infection rates faded three months later, and visitors were once more allowed in hospitals. However, hospital-based restrictions could still be issued if the physical surroundings of a ward or unit did not allow for keeping a two-meter social distance.

### 3.2. Part 2: Cross-Sectional Survey

Out of 88 charge nurses, 71 responded, yielding a response rate of 81%. Nearly all the responding charge nurses were women, and more than half were 50 years or older. They were employed in 29 different departments and primarily from bed wards. Nearly 50% of the charge nurses had more than 10 years of experience as a nurse (Table 1).

#### 3.2.1. Multiple-Choice Questions

During the first and second wave, most often the charge nurses were informed about the current visitor restrictions by the hospital board of directors, their head of department, by regional electronic guidelines, and from the hospital intranet (Table 2). Overall, this information was passed on to the staff at daily meetings and through newsletters and work e-mails. During the first wave, 68% of the charge nurses found the wording of the visitor restrictions clear or very clear and during the second wave it was 86%. One-third of the charge nurses found it challenging to handle the restrictions in practice. During the first wave, 68% of the charge nurses stated that they deviated from the restrictions weekly, or even daily, and during the second wave, it was 74%. No differences in the pattern of responses were found between the first and second waves of the COVID-19 pandemic.

#### 3.2.2. Open-Ended Questions

The qualitative findings complement the results described in Table 2. One of the charge nurses reflected, “We were creating the path while running,” and this specific quote summarizes a wide range of the charge nurses' experiences during the hospital's visitor restrictions, as summarized below.

The charge nurses' doubts about the restrictions were mostly related to insecurity about how best to apply the general restrictions in the specific clinical setting of their unit. During the first wave of the pandemic, they gradually realized that there was room to manoeuvre with supplementary professional considerations to safely deviate from the general restrictions in specific patients' cases. Handling restrictions challenged relations and collaborations both within and outside the ward and required substantial communication efforts from the charge nurses.

Being prepared was a subject commonly mentioned by the charge nurses, in terms of thinking ahead and using virtual and written materials to support communication with patients and relatives. In addition, providing more context-specific information on restrictions was a suggestion for reducing conflicts with relatives. The importance of leadership was evident for the charge nurses, and some highlighted values such as trust, human relations, and common sense in the process of making decisions about visiting.

During the pandemic, it became a basic condition for charge nurses to handle varying visitation restrictions. A clear and concrete information flow was vitally important, and when the information was delayed or not well-synchronised with information given to the public, it caused confusion and disturbances among staff. The charge nurses described how restrictions had both upsides and downsides for both staff and patients. Furthermore, the physical surroundings of the wards limited the possibilities for deviating from restrictions.

## 4. Discussion

Restrictions were communicated in an effective administrative cascade from the Danish Ministry of Health and further on to charge nurses, who led their implementation in each hospital unit. The majority found the wording of the restrictions clear, while one-third found them challenging to handle. Both upsides and downsides to the absence of relatives were identified. To balance the needs of patients, relatives, and staff against the need to prevent the spread of the virus, the charge nurses gradually developed ways to deviate from the restrictions. Communication, collaboration, and leadership were experienced as key tools in the processes of adapting shifting restrictions.

### 4.1. Information Pathways and Communication

The study showed that the information pathway through the Danish healthcare system was considered transparent and coherent during the first year of the pandemic. Along the way, eight shifts in visitor restrictions were made. According to the Organization for Economic Cooperation and Development (OECD), in the OECD Digital Government Index 2023, Denmark was ranked second-best among 38 countries [31]. Also, in the Digital Economy and Society Index 2021, published by the European Commission, Denmark was ranked first [32], indicating a solid digital infrastructure in public governance and that digital skills are generally high among the Danish population. This probably laid the groundwork for the effective digital communication cascade from national guidelines to frontline nursing practices in our hospital. In the current study, the charge nurses found the wording of the visitor restrictions clear, or even very clear. In contrast, Harrison et al. conducted a study scanning policy documents and websites describing COVID-19 adult inpatient visitor restrictions at 70 American medical centres [33]. They found that the information was unclear, inconsistent across the centres, and lacked important details. However, even if the Danish charge nurses generally found the wording of the shifting restriction policies clear, enforcing them was still a complex task when confronted with patients and relatives who perhaps were not informed to the same degree of detail. The charge nurses suggested that should another pandemic occur, the Danish public should be better informed that differing physical surroundings in hospital units would allow differing levels of restrictions.

The importance of communication in handling and implementing the visitor restrictions cannot be overstated. Effective communication is critical to managing any crisis or public health emergency, and COVID-19 has been no exception. In the current study, one-third of the charge nurses found it challenging to manage the visitor restrictions in practice. As shown in Tables 3–5, several challenges were related to communication in the unit and the hospital organization as well as with patients and relatives. In another Danish study, hospital charge nurses with formal management education and leaders with more than five years of experience more effectively managed the COVID-19 situation [13]. In the current study, testing a possible association between years of experience as a charge nurse and their perceptions of the challenges related to managing the shifting visitor restrictions could provide valuable insights. However, the limited sample size of 71 charge nurses precluded such analysis. Therefore, larger studies are needed to investigate this relation.

Leadership has been defined as “… the process of influencing others to understand and agree about what needs to be done and how to do it, and the process of facilitating individual and collective efforts to accomplish shared objectives” [34]. One of the key components in leadership is clear and timely communication. As healthcare environments are complex webs of people and resources, communication processes need to extend upwards and laterally within the organization [35]. Simonovich and colleagues described the importance of effective communication in nursing practice across three levels during the pandemic: organizational leadership, unit leadership, and nurse-to-nurse communication [36]. Furthermore, during the pandemic, the important communication with patients and relatives about the shifting restrictions and their implications for the practice in each unit represented a fourth level of communication. Complex examples of communication-related to all four levels are reflected in the current study. As illustrated, handling the restrictions challenged relations and collaborations both within and outside the ward and required substantial communication efforts from the charge nurses. It is one of many examples that the COVID-19 crisis has presented exceptional challenges for charge nurses.

### 4.2. Managing Shifting Visitor Restrictions

In Denmark during the pandemic, the approach to restrictions changed from being general to more specific and local. When hospitals were allowed to welcome visitors again, it was up to each department to decide the number of visitors allowed, according to their social distancing requirements. At the beginning of the pandemic, from March through May 2020, no visitors were allowed access to the hospitals. In the US, visitor restrictions were more local. Harrison et al. reported that, across 70 medical centres, visitor restrictions varied during a similar period [33]. Seventeen percent did not allow any visitors, and 73% allowed one visitor while the last 10% did not describe the number of visitors allowed. For hospitalised patients with COVID-19, 63% of the centres outlined visitor policies different than those for patients without COVID-19. In contrast, the visitor restrictions in Denmark applied to all hospitalised patients, whether suffering from COVID-19 or not. However, the consistency in visitor restrictions in Denmark was probably due to the size of the Danish healthcare system compared to the American system.

Almost all charge nurses in our study deviated from the restrictions in force. Deviations from the restrictions were wide-ranging in our study, even in an international context. The efficiency of the digital communication cascade from national guidelines to frontline nursing combined with charge nurses' communication and leadership practices may have allowed for a high level of flexibility in managing the visitor restrictions in the individual units. We found the charge nurses deviated from the restrictions to allow relatives of both hospitalised adults and child patients to visit. Across 23 states in the US, exceptions to visitor restriction policies during the COVID-19 pandemic were found in 63 out of 65 hospitals [37]. Setting-specific exceptions included paediatrics, obstetrics/gynaecology, emergency departments, behavioural health, inpatient rehabilitation, surgery, and outpatient clinics. In paediatric units across 36 hospitals in the US, 97% of the units allowed at least one visitor, which underlines how parents are considered the most essential partners in the care of a child [38]. In our study, the charge nurses described exceptions in similar types of settings and further added geriatric wards and intensive care units. Similar results were found in a study of Scandinavian intensive care units [39]. Furthermore, across the different settings, the charge nurses identified patients suffering from a variety of cognitive deficits as one group of patients in particular need of support from their relatives, both when hospitalised and during outpatient treatment. This is an indication that, most likely, all types of clinical settings experienced how the support and involvement of relatives was needed in a wide range of patient pathways; this requires a continued postpandemic nursing focus.

From a societal perspective, deviating from the restrictions to meet complex needs of patients and relatives could be seen as an admirable act of compassion. On the other hand, if deviating could increase the risk of spreading the virus, it could also be seen as a risky practice opposing the general and organizational policies and jeopardizing the health of patients, relatives, and staff. When judging the charge nurses' practice, the relatively low level of total cumulated COVID-19-related deaths in Denmark of 143 per 100,000 people [40] should be considered. Furthermore, as highlighted above, the charge nurses “were creating the path while running.” During the first wave, when knowledge about the disease increased and more protective equipment became available, they gradually learned there could be room to manoeuvre to safely deviate from the restrictions in specific patients' cases.

### 4.3. Supporting Patients and Relatives

Our findings show that, during the serious health crisis of the pandemic, acknowledging the value of family bonds and the role of relatives has been an incentive for the charge nurses to prioritise relatives' presence or to facilitate alternative means of communication. Thus, national restrictions do not seem to capture the complexity at the individual level. Moral distress can occur if an individual is unable to act in accordance with their moral judgment owing to external barriers [41]. The charge nurses reported frequently deviating from the national restrictions as they sought to weigh the interests of the individual patient and his or her relatives against the existing visitor restrictions. Other studies have reported high levels of moral distress among staff witnessing the suffering of patients and relatives who were separated [2]. Obviously, hospitals must ensure the safety of both patients and staff, and the more visitors allowed in the hospital, the more difficult social distancing becomes [42]; however, Valley et al. question whether no-visitor policies are essential for infection prevention at all, and to what extent restricted visitation might unintentionally foster poor patient health outcomes [43].

If visitors are allowed during a pandemic, hospital units need resources to provide personal protective equipment for visitors, and to patients and staff [44]. In Denmark, protective equipment supplies were scarce during the first wave, and staff were not yet fully trained to apply them, likely causing a general reluctance to allow relatives to visit. However, the charge nurses gradually realized there was room to manoeuvre in specific patient cases, if doing so was expected to significantly improve the quality of care. Downar and Kekewich proposed that healthcare organizations adopt a new end-of-life visitor policy that reduces restrictions overall without necessarily putting patients, staff, and family members at an increased risk of COVID-19 transmission [45]. Selman et al. also have recommended advanced care planning that includes regular communication with family members, accurate information provision, support of virtual communication, and enabling family members to say goodbye in person where possible [46]. In the current study, this was what the charge nurses did when adjusting the national restrictions to the local context of their units.

Recent literature on total visitor prohibitions at hospitals during the COVID-19 pandemic describes how palliative care patients, critically ill patients, children, and cognitively impaired patients need alternatives to the support provided by relatives accompanying outpatients to hospital appointments, or when visiting inpatients. Kuntz et al. found that the efficient application of telemedicine for family e-meetings can be feasible and effective for decision making related to dying patients and their families [47]. Selman et al. also recommend applying digital communication strategies to meet the needs of patients and relatives [46]. In a study from an American intensive care unit, a dedicated facilitator helped schedule calls and coordinate virtual communications to reduce the frustrations for patients, family, and HCPs [48]. In our study, the charge nurses would have liked to have been better prepared for virtual communication with relatives, including having the relevant electronic equipment at their disposal. Similar findings have been reported from China [44]. We believe that now, that the pandemic is over, digital communication tools will still be useful to further facilitate the participation of relatives in important hospital conversations if the relative lives far away from the hospital or perhaps is at work at the scheduled time of the conversation.

The charge nurses described how the absence of relatives shed light upon both downsides and upsides to relatives' absence. They also realized that “one size does not fit all.” In the years to come, perhaps these experiences from the pandemic will inspire hospitals with open visiting policies to develop more differentiated visiting policies, welcoming relatives' presence in some units and perhaps limiting their presence in others, to better protect the patients' interests. Nurses may play an important role in balancing the needs of individual patients with the needs of visitors [49]. Nurses' role as a gatekeeper in a flexible visitation practice has also been described in a study of intensive care units [50]. To promote a family-centred approach, it is essential to involve patients and relatives in the process of designing the hospital visiting policies of the future.

### 4.4. Strengths and Limitations

This study has certain limitations. In a document analysis, the selection of documents relies on existing information and may introduce bias as certain materials may be more readily available. Also, the documents may lack the context necessary for a comprehensive understanding [51]. However, the Danish authorities and hospitals constantly updated the population with the most recent announcements. The continuous updating could have brought some difficulties accessing previous information on visitor restrictions that had been adjusted or even changed.

The survey allowed us to collect data from 81% of the hospital's charge nurses. Still, nonresponse bias could occur as the sample did not represent the entire group of charge nurses [52]. As it is a single-site study, the results from the survey cannot be considered representative of all charge nurses in Denmark. Still, as the study was carried out in the largest hospital in the country, the data most likely show some general tendencies. However, the results of the survey may not be generalisable to other situations or settings beyond the research context [53].

The questionnaire was developed for the current study as no existing questionnaire was available. Several initiatives were taken to test the quality of the questionnaire, including double testing of the content of the questions, wording, and layout with several charge nurses, ensuring face validity of the questionnaire [54, 55]. The charge nurses' validation feedback encouraged the authors to include all types of units, especially outpatient clinics and same-day surgery units, which created a more complete dataset. Using face-to-face interviews instead of the electronic questionnaire may have provided more detailed data and in-depth elaboration on the open-ended questions. However, considering the fluctuating infection rates during the study period, this method was not considered feasible. The questionnaire was distributed 14 months after the first wave of the pandemic struck in Denmark. Charge nurses' recollections about the first wave may have induced a systematic error blurred by experiences and behaviours from the second wave causing recall bias [56]. However, the charge nurses seemed to have distinct memories from each of the two waves.

## 5. Conclusion

In Denmark, during the first year of the pandemic, hospital visitor restrictions gradually changed from general to becoming more locally and individually adjusted. A well-organized digital public healthcare information cascade supported the process. Although the information about visitor restrictions was passed through several management levels in the Danish healthcare system, the information generally reached the charge nurses quite effectively.

The charge nurses were informed primarily through other levels of the hospital organization. Even if they generally found the wording of the visitor restrictions clear, one-third found them challenging to handle in practice. Enforcing the restrictions challenged relations and collaborations within and outside the units and required substantial communication efforts from the charge nurses. When making decisions about visiting, trust, human relations, and common sense were highlighted as important leadership values.

The charge nurses played a significant role in balancing the needs of patients, relatives, and staff while managing visitor restrictions during the COVID-19 pandemic. When the charge nurses had to balance general restrictions against the individual needs of patients and relatives, professional nursing values were challenged, and the pandemic has shed light upon downsides as well as upsides to the absence of relatives. Increased knowledge of the disease and the availability of more protective equipment enabled the charge nurses, through professional considerations, to gradually deviate more from the general restrictions in specific patients' cases. Seven out of ten charge nurses deviated from the restrictions weekly or daily.

## 6. Implications for Nurse Managers

In some cases, separation of patients and their closest relative is a paramount burden. When charge nurses deviate from visitor restrictions during a pandemic for the sake of patients and closest relatives, the consequences can extend beyond the immediate healthcare setting with positive as well as potentially negative implications for both the individuals involved and the healthcare institution. The charge nurses' experiences from the pandemic can contribute to further qualifying nurse managers' considerations when designing family-centred hospital visitor policies for the future. Also, they may strengthen the handling of future sudden major organizational changes. Charge nurses require support from both society and hospital managers during a pandemic with visitor restrictions to effectively navigate the challenges they face. This support may come in various forms, including emotional assistance, resource allocation, clear communication strategies, and recognition for their vital role in maintaining healthcare delivery.

## Figures and Tables

**Table 1:** Characteristics of 71 charge nurses.

Characteristics	*n*	(%)
Sex		
Female	67	(94.4)
Male	4	(5.6)
Age, years		
<40	10	(14.1)
40–49	18	(25.3)
≥50	43	(60.6)
Type of ward/unit		
Bed ward	31	(43.7)
Outpatient clinic	17	(23.9)
Short-term unit/Same-day surgery unit	7	(9.8)
Intensive care unit	4	(5.6)
Mixed units or other	12	(16.9)
Experience as a charge nurse, years		
<5	23	(32.4)
5–10	31	(21.1)
>10	29	(46.5)

**Table 2:** Charge nurses' information sources, understanding, handling, and communication of the visitor restrictions.

Research question	Options	First wave		Second wave	
	*N* = 71	*n*	(%)	*n*	(%)
How did you as leader get information about current visitor restrictions?^†^	Hospital board of directors	37	(52)	39	(55)
	Head of department	36	(51)	39	(55)
	Regional electronic guidelines	42	(59)	43	(61)
	Hospital hygiene team	14	(20)	11	(15)
	Hospital intranet (website)	53	(75)	54	(76)
	News media	5	(7)	7	(10)
	Other sources^‡§^	9	(13)	10	(14)

How did you pass on the information about visitor restriction to your staff?^†^	Daily meetings	50	(70)		
	Work e-mails	43	(61)		
	Weekly meetings	17	(24)		
	New letters	52	(73)		
	Posters	24	(34)		
	Other sources^¶^	6	(6)		

How clear was the wording of the visitor restrictions to understand?	Very clear	24	(34)	35	(49)
	Clear	24	(34)	26	(37)
	Unclear	15	(21)	9	(13)
	(Missing)	8	(11)	1	(1)

How did you as leader experience handling the visitor restrictions in practice?	Easy	24	(34)		
	Normal	22	(31)		
	Challenging	24	(31)		
	(Missing)	1	(1)		

How often have you deviated from visitor restrictions paying regard to patients and their relatives?	Never	3	(4)	2	(3)
	Rarely	11	(16)	12	(17)
	Monthly	8	(12)	5	(7)
	Weekly	34	(49)	38	(54)
	Daily	13	(19)	14	(20)
	(Missing)	2	(3)	0	(0)

^†^More than one option was possible. ^‡^First wave: not relevant (*n* = 3), patients and family (*n* = 1), the National Health Authority (*n* = 3), posters (*n* = 1), and other charge nurses (*n* = 1). ^§^Second wave: not relevant (*n* = 3), patients and family members (*n* = 1), the National Health Authority (*n* = 3), other charge nurses (*n* = 1), development charge nurse (*n* = 1), social media (*n* = 1). ^¶^By the department's intranet (*n* = 1).

**Table 3:** Summary of findings from open-ended question 1.

Open-ended question 1: Did you have any doubts about the visitor restrictions, and if so, how were you able to clarify your doubts?	
Category	Illustrative analytic points
Identifying particularly critical situations	(i) Doubts occurred in particularly critical situations related to the care of, for example, dying patients, patients suffering from cognitive deficits, the critically ill, parents of a child or adolescent patient, or young siblings. Also, when staff had to discuss critical treatment issues with a patient, the charge nurses would have preferred relatives to be present.
	(ii) During the pandemic, increased knowledge of the disease and the availability of more protective equipment allowed charge nurses to safely deviate from the restrictions in certain patients' cases

Clarifying doubts through communication	(i) The charge nurses discussed uncertainties with their heads of department, fellow managers, ward staff, patients and relatives, the hospital hygiene team, or the hospital's corona hotline
	(ii) Clarifying doubts was time consuming

Leadership and collaboration	(i) Sometimes the restrictions were managed with variations, causing confusion and frustration among patients, relatives, and staff, such as when a patient was transferred from one ward, department, or hospital to another. This was a challenge to the relationships between staff and patients or relatives and to the collaborations among units, departments, and/or hospitals.
	(ii) The charge nurses found some nurses fully capable of making decisions about deviating from the general restrictions, and they seemed comfortable doing so. Other charge nurses experienced that the nurses wanted the charge nurse to make the decision about visiting in each patient's case, to feel protected by her authority.

**Table 4:** Summary of findings from open-ended question 2.

Open-ended question 2: As a charge nurse, is there anything you would do differently, if a similar situation should occur in the future?	
Category	Illustrative analytic points
Being prepared	(i) The evolving pandemic and the rapidly changing guidelines made the charge nurses feel a step behind things, as leaders. Thus, in a future situation, they wanted to be better at thinking through and planning different scenarios, so they could be ahead of things and reduce stress.
	(ii) The charge nurses would have liked to be better prepared for virtual communication with relatives, including having the relevant electronic equipment at their disposal
	(iii) Further use of posters and information pamphlets explaining the restrictions in specific contexts, such as single/multi-bed rooms, intensive care units, wards, or outpatient clinics would have been helpful

Enforcing precise communication and transparent leadership	(i) Some charge nurses reported that it would have been nice if it had been made more clear to the public that the physical surroundings differ among wards, and consequently wards need to have differing restrictions. Highlighting this information might have reduced conflicts with relatives who did not understand the restrictions.
	(ii) Some charge nurses wondered if an information dissemination task force would have been helpful
	(iii) To avoid unnecessary issues related to interpreting and acting upon the restrictions, the charge nurses described the need for transparent leadership and communication in the organization, both upwards and downwards

Worrying less when deviating from visitor restrictions	(i) The charge nurses had accepted that, when applying visitor restrictions, “one size does not fit all.” After some time, they worried less about making compromises when balancing rules and humanity.
	(ii) Some of the charge nurses suggested trusting the frontline nurses' professional assessments more and using common sense for the sake of the patients

**Table 5:** Summary of findings from open-ended question 3.

Open-ended question 3: Is there anything else you would like to add?	
Category	Illustrative analytic points
Delays in the information flow	(i) Information from the hospital's administrators should be clear and concrete, directing fast production of ward-based guidelines
	(ii) Although charge nurses learned to live with delays in the information flow, it caused anxiety among patients, relatives, and staff, with the latter often having to cope with disturbances that might have been avoided

Upsides and downsides to the absence of relatives	(i) When relatives were not allowed access to the wards, they did not interfere with staff in the timing of their professional work
	(ii) Relatives' absence left the ward environment more tranquil for both patients and staff, which was described as a relief
	(iii) Relatives' absence allowed the patients to rest and recover more
	(iv) Normally, relatives represent a valuable supplementary resource in everyday nurse-patient collaborations, as many things are not possible if the relatives cannot participate
	(v) When adhering strictly to the restrictions, it was difficult for staff to be confronted with the sadness and powerlessness of patients and relatives

The continued managing of social distance requirements	(i) Even when restrictions were loosened, the requirements regarding social distancing still applied, challenging the arrangement of the physical surroundings of the wards

## Data Availability

The data underlying this article can be shared on reasonable request to the corresponding author.
